# Exploring the Potential of Nitrogen-Doped Graphene in ZnSe-TiO_2_ Composite Materials for Supercapacitor Electrode

**DOI:** 10.3390/molecules29092103

**Published:** 2024-05-02

**Authors:** Hassan Akbar, Asghar Ali, Shoaib Mohammad, Faiza Anjum, Ashfaq Ahmad, Amir Muhammad Afzal, Munirah D. Albaqami, Saikh Mohammad, Jeong Ryeol Choi

**Affiliations:** 1 Department of Physics, Abbottabad University of Science and Technology (AUST), Havelian Havelian, Abbottabad 22500, Pakistan; khanhassan849@gmail.com; 2 Pakistan Department of Physics, The University of Lahore, 1-km, Defense Road, Lahore 54000, Pakistan; faizamunzer@gmail.com; 3 Department of Mechanical Engineering, University of Engineering and Applied Sciences, Swat 19201, Pakistan; shoaibkhan@ueas.edu.pk; 4 School of Material Science and Engineering, Shanghai Jiaotong University, Shanghai 200240, China; 5 Pakistan Department of Physics, Riphah International University, Lahore 54000, Pakistan; amir.afzal@riphah.edu.pk; 6 Department of Chemistry, College of Science, King Saud University, Riyadh 11451, Saudi Arabia; muneerad@ksu.edu.sa (M.D.A.); smw.chemistry@gmail.com (S.M.); 7 School of Electronic Engineering, Kyonggi University, Yeongtong-gu, Suwon 16227, Gyeonggi-do, Republic of Korea

**Keywords:** energy storage, ZnSe, TiO_2_, N doped graphene, nanocomposite, supercapacitor

## Abstract

The current study explores the prospective of a nitrogen-doped graphene (NG) incorporated into ZnSe-TiO_2_ composites via hydrothermal method for supercapacitor electrodes. Structural, morphological, and electronic characterizations are conducted using XRD, SEM, Raman, and UV analyses. The electrochemical study is performed and galvanostatic charge-discharge (GCD) and cyclic voltammetry (CV) are evaluated for the supercapacitor electrode material. Results demonstrate improved performance in the ZnSe-NG-TiO_2_ composite, indicating its potential for advanced supercapacitors with enhanced efficiency, stability, and power density. Specific capacity calculations and galvanic charge-discharge experiments confirmed the promising electrochemical activity of ZnSe-NG-TiO_2_, which has a specific capacity of 222 C/g. The negative link among specific capacity and current density demonstrated the composite’s potential for high energy density and high-power density electrochemical devices. Overall, the study shows that composite materials derived from multiple families can synergistically improve electrode characteristics for advanced energy storage applications.

## 1. Introduction

The rapid increase in energy demand, along with the widespread usage of fossil fuels, has given to substantial growth in environmental pollution. The exponential increase in pollution, along with the effects of climate change, threatens the preservation of the ecosystem. These adverse effects significantly harm environmental protections. Furthermore, the issues of energy storage and production are complicated and multidimensional, encompassing everything from technological constraints to economic concerns and environmental consequences. The energy storage industry has multiple challenges related to functional materials, device design, and efficiency in energy conversion. To boost energy storage capabilities, researchers have been exploring innovative materials and composite architectures. Meanwhile, to solve the global energy challenge, researchers are exploring clean and renewable energy sources such as solar, wind, and tidal power [1]. However, these sources are climate-dependent [2]. Therefore, there is an urgent need now to explore and build a long-term and effective energy storage system capable of continuously storing and supplying the needed energy [3]. Developing a high-power output device is a crucial issue for the utilization of renewable energy sources to meet the expanding energy requirements with various portable and electronic devices [4,5,6]. Recently various renewable storage systems, i.e., batteries, fuel cells, and supercapacitors etc. have been used. Supercapacitors (SCs) are very trustworthy for many reasons, including high power density, long-term stability, environmental friendliness, and prolonged charge-discharge features [7,8]. Despite their adaptability, SCs’ limited energy density makes them unsuitable in many applications as energy storages. That limitation prompted researchers to investigate new and more efficient types of supercapacitors with improved performance [9,10,11]. The energy of a supercapacitor (SC) is essentially determined by its operating voltage and capacitance. Researchers have concentrated on increasing electrode material energy density by improving the working electrode capacitance along with its quality [12,13]. The electrode material has a considerable impact on a SC’s performance, with most electrodes made of hydroxides, polymers, transition metal oxides, sulphides, and chalcogenides. Metal-rich chalcogenides, as a kind of electrode material, have garnered significant interest nowadays due to their theoretical capacities (500–1000 mA·h·g^−1^), far exceeding those of commercial graphite anodes (370 mA·h·g^−1^) [14,15,16,17]. Transition metal selenides have higher electrical conductivities than oxide and sulfide ions, due especially to their high volumetric capacitance. Selenium (Se) has found uses in a variety of fields, including strain detectors, surface switches and electronic devices, due to its excellent electrical and chemical stability, and thermal stability [18,19]. Zinc selenide (ZnSe) is known as a good basic material for e^-^ transportation and provides greater mechanical sustenance in heterostructure. Researchers have intensively studied it for electrode material applications [20,21]. Ye et al. produced successfully a NiSe/ZnSe composite material utilizing an in-situ electrodeposition method. The study indicated a great specific capacity of 649.9 mA h g^−1^ at a current density of 1.0 A g^−1^, with a remarkable retentive rate of 98.80% after 10,000 cycles [22]. Similarly, Hussain et al. developed a ZnSe/MnSe composite using an in-situ hydrothermal method for supercapacitor applications. The composite displayed a specific capacitance of 1439.98 F g^−1^ at a high energy density (Ed) of 56.17 Wh kg^−1^ and power density (Pd) 265 W kg^−1^ at 1 A g^−1^ and a preservation capacity of 99.59% after 5000 cycles [23]. Zhu et al. reported the fabrication of CoSe/ZnSe utilizing an in-situ hydrothermal technique for electrode material. The hybrid electrode has a specific capacitance of 92.0 mA h g^−1^ at a current density of 1.0 A g^−1^, with 88.5% retention after 2000 cycles [24]. These investigations demonstrate the use of ZnSe-based composites in the production of high-performance electrode materials for a variety of applications. Furthermore, the nitrogen-doped graphene (NG) has textured as a promising candidate owing to its extraordinary, surface area, electrical conductivity, and adaptable doping levels. Announcing NG into composite materials can produce synergistic effects, follow-on improved charge storage proficiencies [25]. In this context, the incorporation of nitrogen-doped graphene into ZnSe and TiO_2_ composites presents an exciting avenue for advancing supercapacitor technology. Zinc selenide (ZnSe) and titanium dioxide (TiO_2_) are known for their excellent electrochemical properties, including high capacitance and stability. When nitrogen-doped graphene is paired with these materials, the possibility of elevated charge storage capacity is increased along with enhanced ion diffusion kinetics, and improved cycling stability [26,27]. The use of TiO_2_-ZnSe nanocomposites in flexible supercapacitors could produce high energy density. The electrode constructions using NG doped TiO_2_-ZnSe nanocomposites could maximize the accessible surface area and enable ion adsorption and desorption. Furthermore, along with the enhancement of energy density, the capacitance retention could also be improved. The electrochemical capacity of TiO_2_-ZnSe-based supercapacitors is outstanding even when they are exposed to variable mechanical stresses.

In current study, we investigate the characteristics of nitrogen-doped graphene (NG) within the framework of the ZnSe-TiO_2_ composite via hydrothermal method, targeting its application as supercapacitors electrode material. For this purpose, we improve the performance of the composite materials as energy storage by doping NG into TiO_2_, ZnSe, and TiO_2_-ZnSe composites. This work not only resolves the vital aspects of composite design but also frames up the advancement of next-generation supercapacitors, featuring improved efficiency, stability, and power density.

## 2. Results and Discussions

### 2.1. XRD Analysis

**Figure 1:**
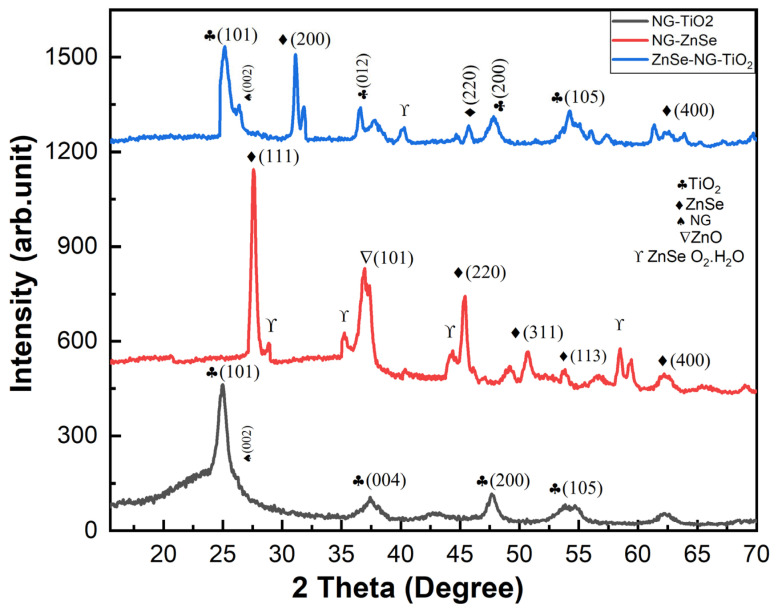
XRD analysis of NG-TiO_2_, NG-ZnSe, and NG-TiO_2_-ZnSe.

The XRD analysis is performed to obseerve the chemical structure of NG-ZnSe, NG-TiO_2_, and NG-TiO_2_-ZnSe composites. The difraction patteren is displayed in Figure 1. The diffraction pattern of NG-TiO_2_ showed (101), (004), (200), and (105) planes, positioned at 2θ = 24.9°, 37.5°, 47.7°, and 54.3° that resemble to tetragonal anatase crystal plane of TiO_2_ [JCPDS No. 21-1272] [28]. Whereas the ZnSe diffraction peaks are indexed as the cubic zinc blende structure which reaches agreement with the XRD data card (JCPDS 37163). The major peaks are appeared at 2θ = 27.6°, 45.5°, and 53.7° and attributed to (111), (220), and (113) planes respectively. However, some additional peaks are appeared due to the interaction between zinc selenide and water molecules [28]. The peaks in the XRD pattern appeared broader, demonstrating a decrease in the crystalline size, owing to the lattice defects by addition of N doped graphene. However, XRD peaks confirms the presence of TiO_2_, ZnSe, and N doped graphene in the composites. Some additional peaks appeared, which might be due to the assimilation of nitrogen atoms in the composites. The spectra for NG-ZnSe composite displayed some additional peaks. The presence of NG may disturb the crystalline characteristics of ZnSe, in comparison with NG-TiO_2_. The interactions between NG and ZnSe could cause lattice strain or chemical bonding that leads to emerging extra diffraction peaks or shoulder peaks. However, during the synthesis of NG doped materials, secondary phases or impurities may also be announced, leading to the appearance of additional peaks in the XRD results. These peaks could line up with or ascend in combination with those of ZnSe, possibly complicating the explanation of the XRD pattern.

### 2.2. SEM

**Figure 2:**
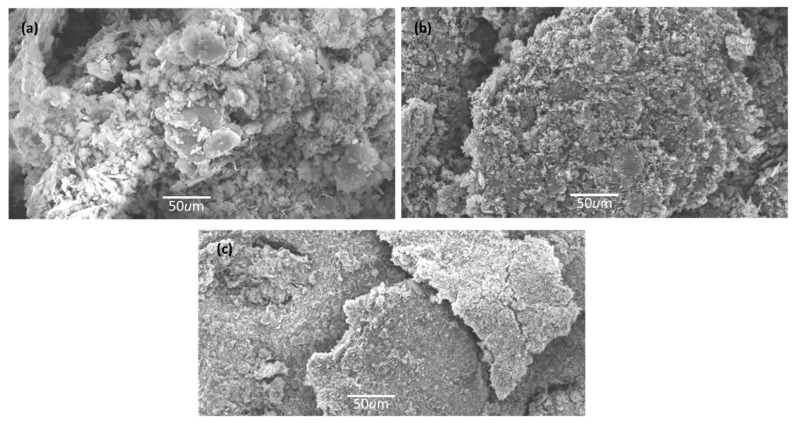
SEM micrographs of N-graphene doped nanocomposite: (**a**) NG-ZnSe, (**b**) NG-TiO_2_, (**c**) NG-TiO_2_-ZnSe.

The morphological characteristics of samples were examined using scanning electron microscopy. Figure 2a–c displays the scanning electron microscope image of NG-ZnSe, NG-TiO_2_, and NG-TiO_2_-ZnSe respectively. The micrograph Figure 2a represented the agglomeration of NG-ZnSe composite. This clustering occurs as a result of interactions between graphene sheets, which create an organized structure. Such patterns are essential for supercapacitor electrodes because they provide a large surface area for ion adsorption and charge transfer processes. Similarly, the NG-TiO_2_ nanocomposite in Figure 2b, displayed an agglomeration of shape like cauliflower. This agglomeration is caused by the van der Waals interactions taken place in graphene sheets, which recompense the bending energy through inter-sheet adhesion, helping to maintain the composite’s stability. The attachment graphene network increases the conductivity of the electrode material, allowing for more effective charge storage and discharge in supercapacitors [29,30,31,32]. In Figure 2c, when NG is doped into TiO_2_-ZnSe composite, the agglomeration is decreased, and the overall surface area of the material is increased. The expanded surface area can moderate the disposition of particles to agglomerate by offering additional sites for interaction and reduced the distance among particles. This less agglomeration may also indicated the enhanced compatibility between materials that reduced interfacial tension and promoted better dispersion [33,34,35].

### 2.3. Raman Analysis

**Figure 3:**
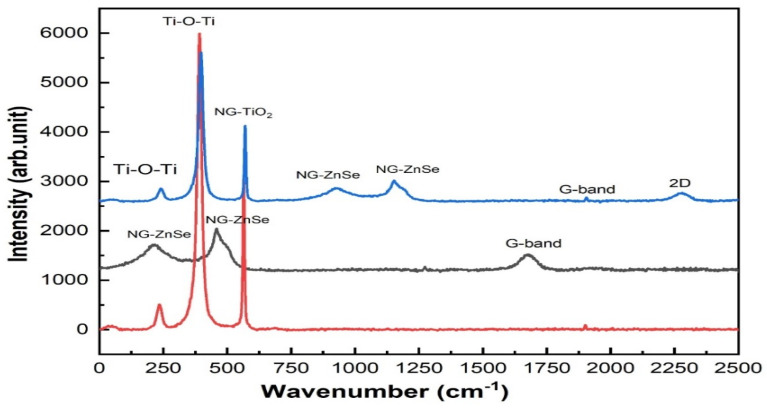
Raman analysis of NG-TiO_2_, NG-ZnSe, and NG-TiO_2_-ZnSe.

Raman spectroscopy is performed to characterize the NG-ZnSe, NG-TiO_2_, and NG-TiO_2_-ZnSe samples, focusing on analyzing the vibrational modes and bonding of the composites. From Figure 3, three broad peaks are observed from the spectrum of NG-ZnSe which are centered about 215, 453, and 1680 cm^−1^. The peaks located at 215 and 453 cm^−1^ are ascribed to the longitudinal optic (LO), transverse optic (TO) and double phonon modes of ZnSe [36,37,38]. The Raman spectra for NG-TiO_2_ is observed at 233, 395, and 568 cm^−1^. The Raman active vibration modes for TiO_2_ observed at 395 (B_1g_) and 568 (A_1g_) can match with the anatase structure of the TiO_2_ [39,40]. The B_1g_ mode specifies the symmetric stretching of the bonds among titanium and oxygen atoms (Ti-O), whereas the A_1g_ is linked with bending vibrations of the Ti-O bonds. The Raman spectrum graph also displayed two prominent bands at about 1625 (G band) and 2278 cm^−1^ (2D band). The D band is attributed to structural defects and disorder, whereas the G band originates from the E_2g_ phonon vibration of sp2 hybridized carbon atoms within the lattice. Commonly the peaks around 1600–1650 cm^−1^ link to the asymmetric stretching vibrations of the N-C bonds in the graphene lattice. The peak associated with N-C vibrations proposed vital insights into the bonding arrangements and the interactions between nitrogen and carbon in nitrogen-doped graphene. These bands serve as primary indicators of the structural integrity and bonding structure of the graphene lattice.

The D band, which appears at around 1625 cm^−1^, represents structural defects and disorder inside the graphene lattice. In supercapacitor applications, structural defects can have a major influence on the material’s performance by affecting electrical conductivity and charge storage capacity. Understanding and measuring these defects is critical for improving electrode material performance and providing long-term stability in supercapacitors. G band, which is caused by the E_2g_ phonon vibration of sp2 hybridized carbon atoms inside the lattice, gives information on graphene’s overall structure and quality. A strong and well-defined G band suggests a high level of graphitization and structural integrity, both of which are desirable properties for supercapacitor electrode materials. Enhanced graphitization increases electrical conductivity and charge transfer kinetics, resulting in greater electrochemical performance in supercapacitors [41,42,43,44].

### 2.4. UV Analysis

**Figure 4:**
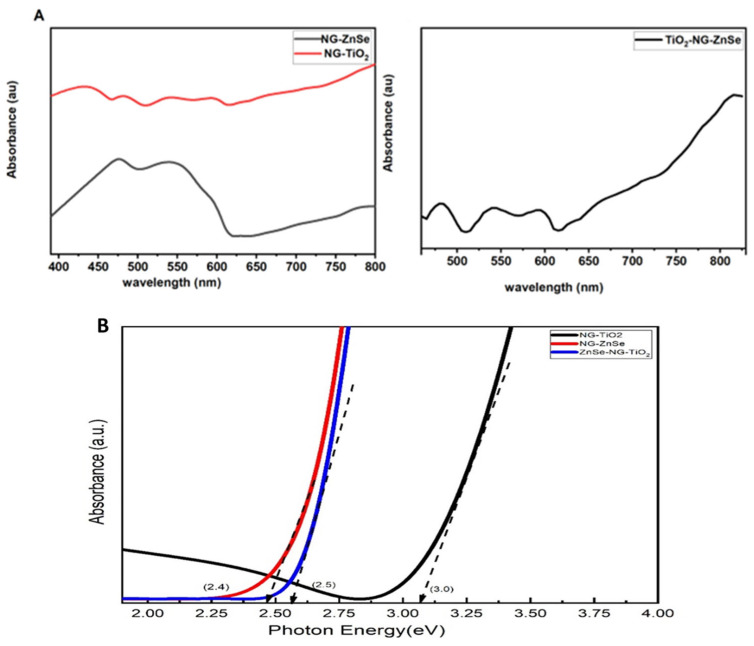
(**A**) Absorption spectra of NG-TiO_2_, NG-ZnSe, and NG-TiO_2_-ZnSe corresponding to sample S1, S2 and S3 respectively. (**B**) Band gap analysis of NG-TiO_2_, NG-ZnSe, and NG-TiO_2_-ZnSe.

The UV analysis is performed to understand the individual bandgaps of the constituent materials after doping N-graphene and their interactions in the composite. Figure 4A represented the absorption spectra of NG-TiO_2_, NG-ZnSe, and NG-TiO_2_-ZnSe corresponding to sample S1, S2, and S3 respectively. The absorption peak of NG-TiO_2_ is found to be arround 410 nm, whereas the absorption peaks of NG-ZnSe and NG-TiO_2_-ZnSe were found 490 and 480 nm respectively. The energy bandgap of the composite is calculated by using tauc plot as shown in Figure 4B. The tauc equation is as follows:

αhv = A(hv − E_g_)^1/2^ (1)

where α is the absorption coefficient, v is the light frequency and E_g_ is the bandgap energy. The bandgap energy of NG-TiO_2_ and NG-ZnSe is calculated as 3.0 and 2.4 eV respectively. The bandgap of NG-TiO_2_ is smaller than the standard bandgap of TiO_2_ (3.25 eV) [45,46], that is due to the dopping of N-graphene, which was also confirmed from XRD and Raman analyses. Similarly the bandgap of NG-ZnSe is lesser than the bandgap of ZnSe (2.7 eV) cited in the literature [47], owing to the doping of N-graphane in ZnSe composite. The bandgap energy (E_g_) of the ZnSe-NG-TiO_2_ composite estimated from tauc plot is 2.5 eV, indicationg that the composite has activity in the UV region. The reduction of bandgap of nanocomposite may offer a room for creating free radicals and fragmentation, those allow the more free electrons on the conduction band. And consequently, for this low value of bandgap, they increase the conductivity of material, enhancing the suitibility of the material for the electronic devices fabrication. The grouping of TiO_2_ and ZnSe along with N-graphene can consequence in the appearance of novel electronic states inside the band gap owing to the interface of their electronic structures. Such intermediate states have the potential to decrease the composite’s total band gap significantly [48,49]. Moreover, at the atomic scale, defects, interfaces, and disorder within the composite material can also impact its electronic properties and contribute to lowering overall band gap [50,51].

## 3. Electrochemistry Performance 

### Cyclic Voltammetry

**Figure 5:**
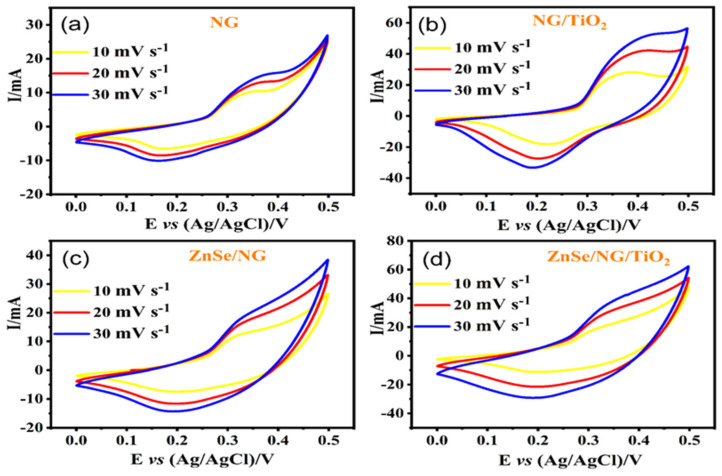
CV characterization; (**a**) NG, (**b**) binary composite of NG/TiO_2_, (**c**) binary composite of ZnSe/NG and (**d**) ternary composite of ZnSe/NG/TiO_2_.

**Figure 6:**
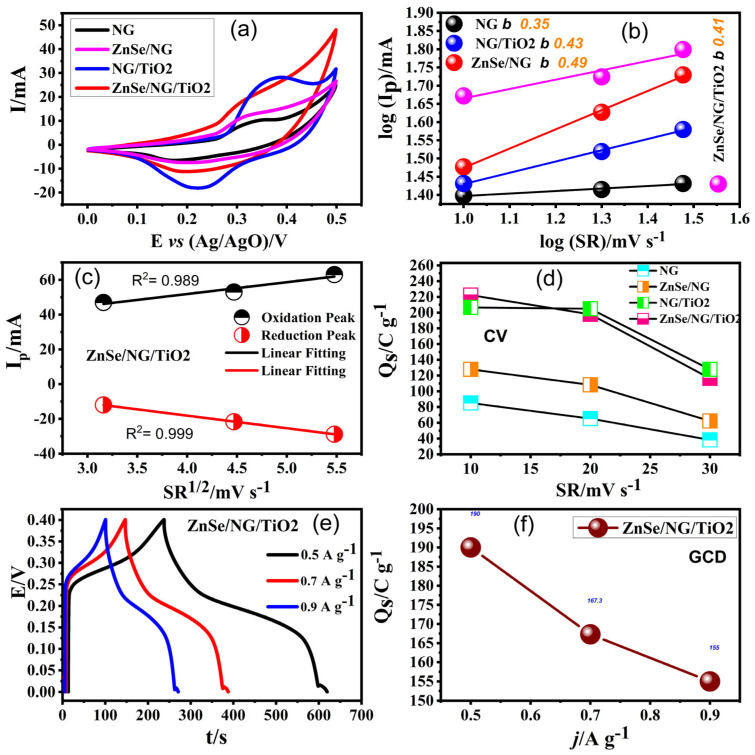
(**a**) Comparison of CV profile of all samples, (**b**) Evaluation of b values for identification of charge storage mechanism, (**c**) Linear fitting relation between oxidation/reduction peak current vs square root of scan rate, (**d**) Dependence of specific capacity on scan rates, (**e**) GCD pattern of ternary composite at various current densities and (**f**) Fluctuation of specific capacity with increase in current density.

To probe the electrochemical prospective of as-synthesized nitrogen-doped graphene and various composites based on it, cyclic voltammetry (CV) and galvanic charge discharge (GCD) characterization were investigated in three electrode assembly combination of Ag/AgCl (Reference electrode), platinum electrode (Counter electrode) and NG, NG/TiO_2_, ZnSe/NG, and ZnSe/NG/TiO_2_ on nickel collectors as working electrodes. Electrochemical profile was analyzed in 6 M KOH electrolyte at constant applied potential of 0.0–0.5 V. CVs measurements for all the given samples have been displayed in Figure 5a–d at different scanning rates; 10 mV s^−1^, 20 mV s^−1^, and 30 mV s^−1^, respectively. All the CVs finger print shows noticeable oxidation and reduction peaks at various scan rates indicating reversible faradic nature of electrode samples. The CV curves clearly indicate that, as the scan rates increase, peak current is raised and the oxidation peak shifts towards higher potential, whereas the reduction peaks swing towards the lower potentials [52,53]. Notably, in Figure 5a, it is evident that the peak current for sample NG is significantly smaller in comparison with other three samples, suggesting a lower electrochemical performance of NG. In Figure 5b the binary compound of NG/TiO_2_ exhibits relatively higher peak currents than pristine NG and good rate capability, while oxidation peaks of NG/TiO_2_ show strong-upraised peak current response at higher scanning rates. Furthermore, in Figure 5c, the CV curves of the binary composite of ZnSe/NG demonstrate superior performance compared to NG, showing improved current retaliation and larger area underneath the curves. The rate capability issue seen in the ZnSe/NG sample can be addressed with the addition of TiO_2_ as clearly. Meanwhile, in Figure 5d, ternary composite ZnSe/NG/TiO_2_ reveals outstanding electrochemistry behavior among all the tested samples in terms of faradic activity, rate capability, and superior integrated area under the CV curves which all are considered for superior performance of electrode for efficient energy storage applications. The comparison of all the above composites CV profile is shown in Figure 6a.

The bond among anodic peak current (*i_p_*) and scan rate (*v*) is analyzed by means of a power law equation [54,55]:

(2) $$i_{p}=av^{b}\;\;\;\;\;\;\;or\;\;\;\;\;\;\;logi_{p}=loga+blogv$$

where ‘*a*’ and ‘*b*’ are adjustable variables. Usually, the power-law exponent *b*, is expected to range between 1 and 0.5. An exponent value close to 1 signifies capacitive behavior (surface-controlled), while around 0.5 indicate a dispersion–restricted progression (inner surface controlled). The plot of log(*i_p_*) against log(*v*) in Figure 6b yields a slope of 0.35, 0.43, 0.49, and 0.41 all close to 0.5, demonstrating the dispersion control charge process in the ternary composite of NG, NG/TiO_2_, ZnSe/NG, and ZnSe/NG/TiO_2_ [56].

Further investigation of ZnSe/NG/TiO_2_ involves employing a theoretical approach. The anodic and cathodic peaks of the current are linearly fitted (depicted in Figure 6c) the steady fit of peak currents versus the square root of scan rate demonstrates, lower shift of peaks and higher shift of peaks of voltage with increasing scan rate. The ultimate current exhibits a relationship relative to scan rate square $\sqrt{v\;(mV/s)}$, indicated the high value of R^2^ for both anode and cathode peaks to be 0.989 and 0.999 correspondingly. The nearly equal values indicate their stability, ZnSe/NG/TiO_2_ as an electrode material, supporting the reversibility of the redox reactions. Furthermore, to reinforce our position regarding the favorable electrochemical activity of ZnSe/NG/TiO_2_ composite material, specific capacity *Q_s_* is determined based on the CV plot. To achieve this, specific capacities are calculated utilizing Equation (2), and the resulting data is depicted in Figure 6d across all corresponding scan rates [57,58,59]. The equation for specific capacity is of the form

(3) $$Q_{s}=\frac{1}{2mv}\int I\times VdV$$

(4) $$Q_{s}=\frac{I\times t}{m}$$

In these equations, *m*, *v* and ∫I×VdV present active mass of electrode material on nickel foam, scan rate, and area under CV curves respectively. Similarly, *I* and *t* show charging-discharging current in mA and discharge time in seconds. The specific capacity founded for NG is 85.2 C/g, while the specific capacities for other composites NG/TiO_2_, ZnSe/NG, and ZnSe/NG/TiO_2_ are 207, 128, and 222 C/g, respectively. In particular, the specific capacity performance of ZnSe/NG/TiO_2_ is promising and this beneficial aspect is ascribed to merging of individual characteristics of NG, ZnSe, and TiO_2_, which reinforces each other in composite matrix [25,60,61,62]. To further elucidate the advantageous feature of ZnSe/NG/TiO_2_ composite for electrochemical energy storage systems, GCD test was scrutinized in three electrode assembly of 6 M KOH solution applying potential window of 0–0.4 V at current densities of 0.5 A g^−1^, 0.7 A g^−1^, and 0.9 A g^−1^, respectively. The narrower 0.4 V potential window for GCD was selected on a specific voltage regime and specifically considered where particular electrochemical phenomena, such as ion insertion/extraction or capacitive behavior, are expected to be dominate. GCD pattern of ternary composite shows nonlinear behavior with respect to charge-discharge time indicating diffusion-controlled charge storage mechanism of the electrode material portrayed in Figure 6e. Moreover, it is evident from Figure 6e, that escalating current densities results in a reduction in discharging time, primarily due to insufficient time for electrolyte diffusion. Nonetheless, it is striking that at lower current densities, the discharging time increases, attributed to the adequate duration provided for the diffusion of OH^−1^ ions into the bulk phase of ZnSe/NG/TiO_2_ composite [60,61,63,64,65,66]. The specific capacity obtained for GCD plot is 195 C g^−1^ by using Equation (4). This value has good agreement with the obtained value for specific capacity in CV test, thereby enabling us to explore better utilization of ZnSe/NG/TiO_2_ composite for potential electrode performance. Further, the relationship curve is developed between specific capacity and current density in Figure 6f, describing the inverse nature with each other. For this study, we concluded that further investigation are underway focusing on the conformation and morphology of conducting ternary composite for use as electrode materials. These investigations are driven by the pursuit of meeting the demanding criteria for high performance energy storage systems.

**Figure 7:**
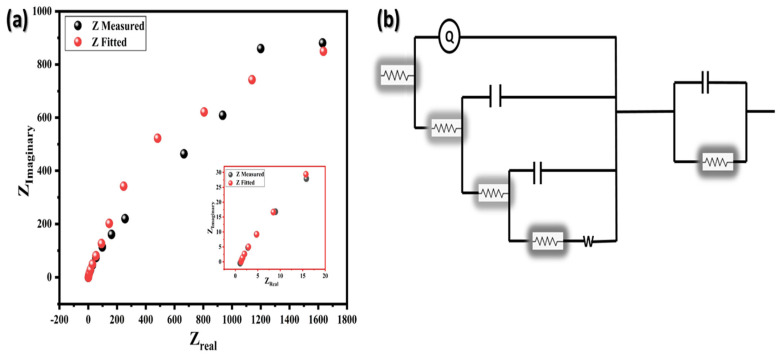
(**a**) Nyquist plot of EIS ZnSe/NG/TiO_2_ ternary composite electrode. The inset EIS shows at the bottom and (**b**) Rundles equivalent circuit at top corner.

To evaluate the charge transfer resistance of the composites material that has been prepared, EIS is recorded as illustrated in Figure 7, in 6 M KOH solution at the frequency range from 10^6^ to 10^−1^ Hz at 0.0 V (vs. Ag/AgCl). In Figure 7a, the initial segment of intersection between the arc depicted in the Nyquist plot and the real impedance (Z_real_) implies the bulk electrolyte resistance (R_e_) or the equivalent series resistance (ESR) set within the high-frequency region. Meanwhile, the intermediate section of the arc denotes the charge transfer resistance (R_CT_), situated within the intermediates frequency range. The lesser the arc diameter, less will be the charge transfer resistance, allowing the more charge transfer in electrolyte mechanism. This effective separation is caused by incorporation of N doped graphene and addition of ZnSe with TiO_2_. The fitting of EIS spectra based on the Rundles equivalent circuits is shown in the inset of Figure 7b. The quasi-linear EIS profiles observed in the lower frequency range indicate the presence of Warburg impedance, arising from the frequency-dependent diffusion/transport of ions within the electrolyte. 

## 4. Experimental Work

### 4.1. Material Preparation

The N dopped graphene was added to ZnSe and TiO_2_ powder by hydrothermal method. For this purpose, N-graphene was prepared at frist. Graphene oxide (GO) was taken as a precursor for the synthesis of N-graphene. Hydrothermal method was used to introduce nitrogen dopants into the graphene lattice. The GO (100 mg) suspension was mixed with (30 mL) deionized water (ultrasonicate at 30 min), and the nitrogen precursor (10 mL of Ammonia solution and 10 mL of N_2_H_4_·H_2_O) was further added into that GO solution, followed by stirring of it for 20 min. After that the mixture was shifted to a sealed Teflon autoclave at high temperatures (180 °C) for 12 h. This condition promoted the reduction of GO and the integration of nitrogen dopants into the graphene lattice. Then vessel was allowed to cool down at room temperature. The nitrogen-doped graphene material was rinsed multiple times with distilled water to eradicate any lingering reactants or impurities. After preparing N-graphene (20 mg), it was dopped into ZnSe (20 mg) and TiO_2_ (20 mg) composite by (1:1:1) ratio using again hydrothermal methods. Three samples, NG-ZnSe, NG-TiO_2_, and ZnSe-NG-TiO_2_, were prepared. For sample 1, the ZnSe compsite was gradually introduced into the GO solution, whereas this causes immediate sedimentation due to electrostatic interactions between the composite and GO. The mixture was then magnetically stirred at 25 °C for 5 min before being transferred to a 50 mL autoclave. Subsequently, the deposits tolerated a hydrothermal process at 180 °C for 12 h, resulting in the formation of NG-ZnSe nanocomposites. This straightforward approach was also applied to prepare other two samples [67,68,69].

### 4.2. Electrode Preparation 

The electrodes were prepared to perform electrochemical analysis. For this purpose the 15% activated carbon black, 5% polyvinylidene fluoride (PVDF), and 80% active composite by weight were added in N-methyl pyrrolidone (NMP). The mixture called slurry was sonicated for 30 min. The Ni foam was prepared to deposit the composite material. It was washed to remove the oxides and imperfections. To conduct this cleansing process, a 1 M HCl solution was created by diluting it with 50 mL of deionized water. The Ni foam washed with HCl trailed by bathing with deionized water and then dryed. After washing process, the slurry was deposited on Nickel (Ni) to make electrode for assessing electrochemical properties. Each electrode had approximately 3 mg/cm^2^ of active material (slurry) on the substrate. This slurry was uniformly applied onto the Ni foam and then the prepared electrodes were dried at 60°C for 8 h. The electrochemical performance was evaluated through tests of the individual electrodes by means of a three-electrode setup at 25 °C, in 6.0 M KOH as the appropriate electrolyte. The setup included one Ag/AgCl reference electrode and a Pt wire counter electrode.

### 4.3. Characterization

The structural analysis of the samples was performed using X-ray diffraction (XRD) with a Cu Kα radiation (λ =  0.15406 nm) within the 0°–70° range. The morphology of samples was analyzed using a field emission scanning electron microscope (FESEM). The sample’s structure was inspected applying Raman spectroscopy. The sample’s absorption range and properties were assessed through ultraviolet-visible diffuse reflectance spectroscopy (UV-VIS DRS). Galvanostatic charging and discharging (GCD), Cyclic Voltammetry (CV),and Electrochemical Impedence Spectroscopy (EIS) experiment were conducted using an Origaflex OGF500system (Origalys, France).

## 5. Conclusions

In conclusion, the incorporation of nitrogen-doped graphene into ZnSe and TiO_2_ composites for super capacitor electrodes was successfully explored in this study. The structural and morphological analyses confirmed the presence of desired materials in the composites. Raman spectroscopy provided insights into vibrational modes and bonding arrangements. UV analysis revealed reduced band gaps after NG doping, indicating improved electronic properties. Electrochemical evaluations through cyclic voltammetry and galvanostatic charge-discharge demonstrate the superior performance of the NG-TiO_2_-ZnSe composite. The ternary composite exhibits diffusion-controlled charge storage, high rate capability, and stable redox reactions. The specific capacity values support the composite’s potential for advanced high-energy-density and high-power-density electrochemical devices. Overall, the study lays a foundation for the development of next-generation supercapacitors with enhanced characteristics.

## Data Availability

The data supporting the findings of this study are not publicly available. However, they are available from the corresponding author upon reasonable request.
